# Hyaluronan Mediates Cold-Induced Adipose Tissue Beiging

**DOI:** 10.3390/cells13151233

**Published:** 2024-07-23

**Authors:** Xi Chen, Yifan Wang, Huiqiao Li, Yanru Deng, Charlise Giang, Anying Song, Yu’e Liu, Qiong A. Wang, Yi Zhu

**Affiliations:** 1 USDA/ARS Children’s Nutrition Research Center, Department of Pediatrics, Baylor College of Medicine, Houston, TX 77030, USA; 2 Department of Molecular Endocrinology, Diabetes and Metabolism Institute, City of Hope Medical Center, Duarte, CA 91010, USA; 3 Tongji University Cancer Center, School of Medicine, Tongji University, Shanghai 200092, China

**Keywords:** hyaluronan, cold-induced adipose tissue beiging, extracellular matrix, HAS2, PH20

## Abstract

Adipose tissue beiging refers to the process by which beige adipocytes emerge in classical white adipose tissue depots. Beige adipocytes dissipate chemical energy and secrete adipokines, such as classical brown adipocytes, to improve systemic metabolism, which is beneficial for people with obesity and metabolic diseases. Cold exposure and β3-adrenergic receptor (AR) agonist treatment are two commonly used stimuli for increasing beige adipocytes in mice; however, their underlying biological processes are different. Transcriptional analysis of inguinal white adipose tissue (iWAT) has revealed that changes in extracellular matrix (ECM) pathway genes are specific to cold exposure. Hyaluronic acid (HA), a non-sulfated linear polysaccharide produced by nearly all cells, is one of the most common components of ECM. We found that cold exposure significantly increased iWAT HA levels, whereas the β3-AR agonist CL316,243 did not. Increasing HA levels in iWAT by *Has2* overexpression significantly increases cold-induced adipose tissue beiging; in contrast, decreasing HA by *Spam1* overexpression, which encodes a hyaluronidase that digests HA, significantly decreases cold-induced iWAT beiging. All these data implicate a role of HA in promoting adipose tissue beiging, which is unique to cold exposure. Given the failure of β3-AR agonists in clinical trials for obesity and metabolic diseases, increasing HA could serve as a new approach for recruiting more beige adipocytes to combat metabolic diseases.

## 1. Introduction

“Beige” or “brite” adipocytes, which reside in white adipose tissue (WAT) depots, dissipate energy as heat and secrete adipokines to regulate systemic metabolism in mice [1,2,3]. These adipocytes, together with classical brown adipocytes, play an important role in energy balance and possess great potential for treating metabolic diseases [4,5,6]. The abundance of brown adipocytes is low in human adults, but can be significantly induced under cold temperature, chronic stress, or drug treatments [7,8,9,10,11,12,13]. These induced thermogenic adipocytes possess a transcriptomic signature similar to that of mouse beige adipocytes [14]; thus, studies on mouse beige adipocytes and the processes used to induce them, referred to as “beiging”, have tremendous implications for human health. Cold and β3-adrenergic receptor (AR) agonist treatments are two commonly used stimuli for increasing beige adipocytes in mice, but the underlying mechanisms are different. Cold conditions predominantly promote the recruitment of new beige adipocytes, also known as de novo beige adipogenesis [1], while β3-AR agonists mainly promote the conversion of white adipocytes to beige adipocytes [15,16]. Clinical trials testing β3-AR agonists for obesity showed minimal efficacy, while highlighting safety concerns [17,18,19], likely due to significantly lower β3-AR expression in human adipocytes [20,21]. Thus, understanding how cold distinctly recruits beige adipocytes is particularly important for the development of therapeutics to increase thermogenic adipocytes and energy expenditure in humans.

Comparative transcriptome profiling revealed that genes in the extracellular matrix (ECM) pathway are most significantly affected (suppressed) by cold, but not by β3-AR agonist CL316,243 treatment in inguinal white adipose tissue (iWAT) [22]. ECMs are composed of glycosaminoglycans (e.g., hyaluronate), proteoglycans, and glycoproteins [23], providing structural support, as well as a biochemical and biomechanical environment for enmeshed cells [24]. Sodium hyaluronate is the sodium salt of hyaluronic acid (HA), a non-sulfated linear polysaccharide produced by almost all cells [25,26,27,28]. We found that cold, but not the β3-AR agonist CL 316,243, increases iWAT HA content, implicating a unique role of HA in cold-induced adipose tissue beiging.

HA is synthesized from uridine diphosphate (UDP)-glucuronic acid (UDP-GlcUA), and UDP-N-acetylglucosamine (UDP-GlcNAc), both derived from glucose. It was initially thought that obese and type 2 diabetes patients with high blood glucose levels would produce more HA, impair insulin sensitivity, and contribute to the pathogenesis of the disease. In our previous study, we found that despite an increase in serum HA levels, adipose tissue HA content decreased after prolonged high-fat, high-sucrose (HFHS) obesogenic diet treatment [29]. Using an inducible, adipocyte-specific transgenic model, Adipoq-rtTA::Tre-Has2, abbreviated as Apn-Has2 mice, we found that increased adipose tissue HA production significantly improved systemic glucose tolerance without affecting systemic insulin sensitivity [29]. When Apn-Has2 mice were acutely challenged by cold exposure in the TSE metabolic chamber, they produced more heat (indirectly calculated from oxygen consumption), even though their brown adipose tissue UCP1 expression did not differ significantly from that of the control mice at the beginning of the “cold challenge”, when mice were housed at room temperature [29].

These data enabled us to carry out long-term cold exposure, which demonstrated that *Has2* overexpression in adipocytes enhances cold-induced adipose tissue beiging, whereas digestion of HA in a new mouse model of hyaluronidase PH20 overexpression dampens cold-induced adipose tissue beiging. Altogether, these data suggest a positive role for HA in the cold-induced beiging process of adipose tissue. To identify the mechanisms that govern the effect of HA in cold-induced adipose tissue beiging, we performed RNA sequencing of the iWAT depots from Apn-Has2 and Apn-Spam1 mice, which revealed significant changes in the ECM pathway, as expected, as well as changes in chemotaxis and growth factor pathways. We discuss exactly how HA promotes adipose tissue beiging and why this effect is unique to cold exposure, which warrants further research in the future.

## 2. Materials and Methods

### 2.1. Mice

Animals were cared for and used in accordance with the United States Animal Welfare Act, and the experimental protocols were approved by the Institutional Animal Care and Use Committee of Baylor College of Medicine. Adiponectin-rtTA mice (referred to as Apn-rtTA) were previously generated, verified, and gifted by Dr. Philipp Scherer [1]. TRE-Has2 and TRE-PH20 mice were generated by subcloning the respective mouse cDNA gene into the pTRE vector (Clontech, Mountain View, CA, USA) with a rabbit β-globin 3′-UTR [30]. All experiments were conducted using littermate-controlled male mice starting at 8–10 weeks of age unless mentioned otherwise. Mice were housed in cages with a 12 h dark–light cycle and free access to water and regular chow or a chow diet containing 200 mg/kg doxycycline (Dox200; Bio-Serv, S3888, Flemington, NJ, USA). Mice were randomly allocated to experimental groups and weight-matched at the beginning of the experimental protocol. For the cold-induced beiging experiments, mice were singly housed in temperature-controlled chambers at 6 °C for the indicated periods with free access to food and water. CL 316,243 (Sigma-Aldrich, C5976-5MG, St. Louis, MO, USA) was administered at a dose of 1 mg/kg body weight via intraperitoneal injection.

### 2.2. Stromal Vascular Fraction (SVF) Isolation and Treatment

Mice were euthanized, and iWAT was dissected and minced into small pieces. The minced tissue was incubated in a digestion buffer containing 1 mg/mL collagenase D at 37 °C for 90–120 min to break down the extracellular matrix and dissociate the adipocytes and SVF cells. Subsequently, the digested tissue was passed through a 100 µm mesh, followed by centrifugation to remove the floating adipocyte fraction. The resulting pellet was then resuspended and further filtered through a 40 µm mesh to remove any remaining undigested fragments. The filtrate was centrifuged at 600× *g* for 5 min to pellet SVF cells. The SVF pellet was resuspended in 5 mL DMEM/F12 medium (Thermo Fisher, 11330057, Waltham, MA, USA) supplemented with 10% fetal bovine serum (Sigma-Aldrich, F0926-500ML, St. Louis, MO, USA), 1% penicillin/streptomycin (Sigma Aldrich, P4333-100 ml), and 0.1% gentamycin (Thermo Fisher, 15-710-064) and then seeded in a 60 mm plate. Once the cells reached 80–90% confluence, they were seeded into a 12-well plate. SVF cells were treated with 110 nM CL316,243 (Sigma-Aldrich, C5976; EC50 = 3 nM) or 500 nM isoproterenol (Cayman Chemical Company, 15592; EC50 = 45.6 nM, Ann Arbor, MI, USA) for 4 h. Both doses were expected to achieve full activation of respective β-ARs. Following this incubation, the medium was collected for HA quantification.

### 2.3. RNA Extraction and qPCR

Frozen iWAT tissue was homogenized in TRIzol (ThermoFisher, 15596018) to disrupt the cells and release RNA. Chloroform (Fisher Scientific, C298-500, Hampton, NH, USA) was added to the homogenate to create a biphasic solution that separated RNA from DNA and proteins. The mixture was centrifuged to separate the phases; the top lipid layer was removed, and the aqueous phase containing the RNA was transferred to a new tube, precipitated, and centrifuged to obtain the RNA pellet. Subsequently, the RNA pellet was washed with ethanol to remove any contaminants. The purified RNA pellet was then resuspended in RNase-free water. Finally, the quantity and quality of the isolated RNA were assessed by spectrophotometry; 1 mg of RNA was used to transcribe cDNA using a reverse transcription kit (Bio-Rad, 1708841BUN, Hercules, CA, USA). RT-qPCR primers were obtained from Harvard PrimerBank [31] (https://pga.mgh.harvard.edu/primerbank/) (accessed between 1 January 2020 and 1 July 2024) (Table 1). The mRNA levels were calculated using the comparative threshold cycle (Ct) method and normalized to the gene *Rps16*.

### 2.4. RNA Sequencing

RNA was extracted according to the previously described protocol. The concentration of the RNA samples was measured using a NanoDrop Spectrophotometer (Thermo Fisher), and their integrity was confirmed using an Agilent 2100 Bioanalyzer (Agilent Technologies, Santa Clara, CA, USA). Only samples with RNA integrity numbers (RINs) above 8.0 were utilized in the experiments. cDNA libraries were constructed using the Illumina TruSeq RNA Sample Preparation Kit (Illumina, San Diego, CA, USA), resulting in an average cDNA library size of 150 bp (excluding adapters). The quality and integrity of these cDNA libraries were verified using an Agilent 2100 Bioanalyzer (Agilent Technologies) and ABI StepOne Plus real-time PCR system (Thermo Fisher). RNA sequencing was performed by Novogene (Novogene, Sacramento, CA, USA). FastQC v0.11.9 was employed to check the raw sequencing quality and test for adapter contamination. The overall quality was satisfactory, and the raw reads were mapped to the mouse genome using STAR v2.7.9a. The genome index was generated from raw FASTA, and annotation files were downloaded from the GENCODE portal for the mouse genome build GRCm38 release 23. Alignments were stored in the BAM format. MultiQC v1.12 provides a summary of the raw read and alignment quality. Gene expression values for each sample were calculated based on the number of reads aligned per gene using STAR quantMode GeneCounts. Raw counts were normalized, and genes with an average read count below 50 across all samples were excluded from differential analysis. Differential gene expression analysis was performed using DESeq2, applying a *p*-value threshold of 0.05 to identify significant differentially expressed genes. 

### 2.5. HA Extraction and Measurement

HA extraction was performed as previously described [32]. The harvested serum samples were directly used for HA measurement. For iWAT, the tissues were excised immediately after the animals were sacrificed and weighed. Approximately 20 mg of iWAT was homogenized using a TissueLyser in homogenization buffer containing 10 mM Tris-Cl (ThermoFisher, 15567027), 5 mM EDTA (Boston Bioproducts, BM-150-K, Milford, MA, USA), 100 mM NaCl, and 0.1% SDS with proteinase K (Sigma-Aldrich, 3115879001) and then digested at 55 °C overnight. A total of 0.8 M MgCl_2_ (Sigma-Aldrich, M1028-100ML) was added to chelate EDTA, followed by incubation at 95 °C for 10 min to inactivate the proteinase K. The digested mixture was centrifuged to remove the indigestible tissues, and Pefabloc SC (Sigma-Aldrich, 11873601001) was added to the collected supernatant to inhibit residual proteinase K activity. The samples were incubated at 37 °C overnight. Subsequently, 0.5 μl of benzonase endonuclease (Sigma-Aldrich, E1014-5KU; >125 IU) was added and incubated at 37 °C overnight to remove nucleic acids. The samples were then incubated at 65 °C for 20 min to inactivate benzonase endonuclease. Pure ethanol (Fisher Scientific, BP28184) was added, and the samples were incubated at −80 °C overnight to precipitate HA. The samples were then centrifuged to collect the HA pellet, which was washed once with 76% ethanol. Finally, the HA pellet was dissolved in 10 mM Tris-Cl (pH 8.0) for the HA ELISA.

For HA measurement, the extracted HA was quantified using an ELISA kit (R&D Systems, DHYAL0, Minneapolis, MN, USA) with a sample digested with bovine testis hyaluronidase overnight as the negative control. Specific dilutions of the samples were predetermined to allow the final readings to fall within the linear portion of the transformed standard curve. Diluted samples were measured using a manufacturer-provided protocol. The tissue HA content was expressed as measured HA levels normalized to the wet weight of the tissue used for extraction.

### 2.6. Histology

After euthanasia, the tissue was immediately excised, fixed overnight in 10% PBS-buffered formalin, and stored in 50% ethanol. Tissues were sectioned (5 µm), rehydrated, and stained with hematoxylin and eosin (H&E) at the Pathology Core of BCM. Microscopic images were obtained with a ZEISS Axioscan scanner (ZEISS, Oberkochen, Baden-Württemberg, Germany).

### 2.7. Immunofluorescence

Paraffin sections were first deparaffinized and rehydrated by sequentially washing in xylene (Sigma Aldrich, 534056-4L), 100% ethanol, 95% ethanol, and distilled water. The slides were boiled in 10 mM sodium citrate buffer (VectorLab, H-3300-250, Newark, CA, USA) for 10 min to unmask the antigens. After cooling on the benchtop for 30 min, the samples were rinsed twice with TBST. The specimens were blocked in blocking buffer (2% BSA in TBS with 0.5% Tween-20) for 60 min and then incubated with UCP-1 primary antibodies (CST, 72298) in antibody dilution buffer (2% BSA in TBST) overnight at 4 °C. After rinsing three times in TBST for 5 min each, the specimens were incubated with fluorochrome-conjugated secondary antibodies (Invitrogen, A32734, Waltham, MA, USA) for 1–2 h at room temperature in the dark. After three more washes in TBST, the specimens were incubated with 300 nM DAPI (ThermoFisher, D1306) solution for 5 min at room temperature, rinsed, and mounted using Dako mounting medium (Aligent Technologies, S3023). Microscopic images were obtained using a BC43 confocal microscope (Oxford Instruments, Abingdon, UK). The mages were acquired under the same parameters, and overall channel immunofluorescent signal intensity was quantified using images containing an average of 100–500 adipocytes and the Image J software version 1.54g.

### 2.8. Statistical Analysis

Results are shown as the mean ± SEM. Student’s *t*-test was used to compare the difference between the two groups, and one-way ANOVA was used to assess differences among the three groups, followed by Tukey’s multiple comparison test for pairwise comparisons between the groups. *p* values < 0.05 were considered statistically significant. Detailed statistical analyses and significance levels are provided in the figure legends.

## 3. Results

### 3.1. Cold, but Not CL 316,243, Increases iWAT HA Levels

**Figure 1:**
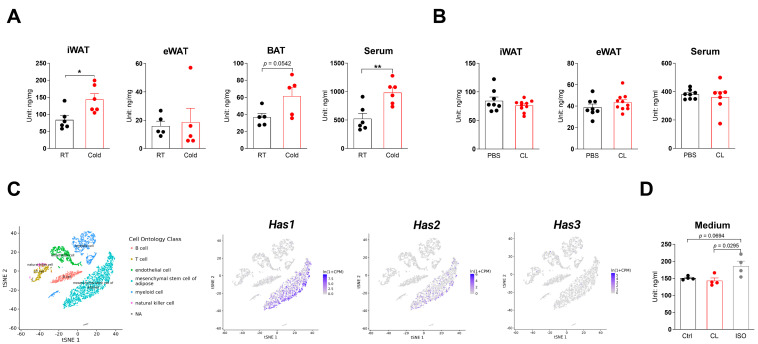
Cold, but not CL 316,243, increases iWAT HA levels. (**A**) HA contents in inguinal white adipose tissue (iWAT), epididymal white adipose tissue (eWAT), brown adipose tissue (BAT), or serum after overnight cold exposure. Two cohorts of mice were used. iWAT and serum were collected from cohort 1, *n* = 6; eWAT and BAT were collected from cohort 2, *n* = 5. (**B**) HA contents in iWAT, eWAT, or serum from mice treated with 1 mg/kg body weight CL 316,243 treatment overnight. iWAT and eWAT were collected in one cohort of mice, with *n* = 8–10; sera were collected from a separate cohort of mice with *n* = 7–8. (**C**) Expression of *Has1*, *Has2*, and *Has3* genes in flow-sorted cells in the white adipose tissue, plotted using data from Tabula Muris database. (**D**) HA levels in cell culture medium of stromal vascular fraction cells subjected to 110 nMCL 316,243 (CL) or 500 nM isoproterenol (ISO) treatment. All data are mean ± SEM. * *p* < 0.05, ** *p* < 0.01.

Sex and age affect HA levels in circulation and iWAT (Supplementary Figure S1); therefore, we chose to begin our study using young male mice. Overnight cold exposure (6 °C) of 10-week-old male mice increased iWAT HA levels by 79% and increased serum HA levels by 87.5%, while having a minimal effect on epididymal WAT (eWAT) HA levels (Figure 1A). Cold exposure also increased brown adipose tissue (BAT) HA levels by 68%, but this difference was not statistically significant (Figure 1A). In contrast, overnight treatment with 1 mg/kg body weight CL 316,243 via intraperitoneal (i.p.) injection did not significantly affect HA content in iWAT or serum HA content (Figure 1B). This dose of CL 316,243 has been well established to induce adipose tissue beiging [33]. In addition to adipocytes, adipose tissue comprises adipocyte progenitor cells, blood vessel endothelial cells, and immune cells. Plotting the published adipose tissue cell population expression data revealed that mesenchymal stem cells (MSCs) of adipose tissue had the highest expression of Has genes, with Has1 expressed in the highest percentage of cells and Has3 expressed in the lowest percentage of cells at a nearly nonexistent level (https://tabula-muris.ds.czbiohub.org/; accessed on 16 June 2024 Figure 1C). Cold exposure promotes the secretion of norepinephrine (NE), which can function on all three β-ARs. A cold-mediated but not β3-AR-mediated process can be mediated through β1-AR, β2-AR, or both. Treatment of isolated stromal vascular fraction (SVF) cells, which comprise adipose progenitor cells (APCs) but not adipocytes, with the β3-AR agonist CL316,243 or pan-β1/2-AR agonist isoproterenol (ISO) showed selected increases in culture medium HA in the ISO group (Figure 1D), indicating that β3-AR does not participate in promoting HA production. 

### 3.2. More HA Promotes Adipose Tissue Beiging

**Figure 2:**
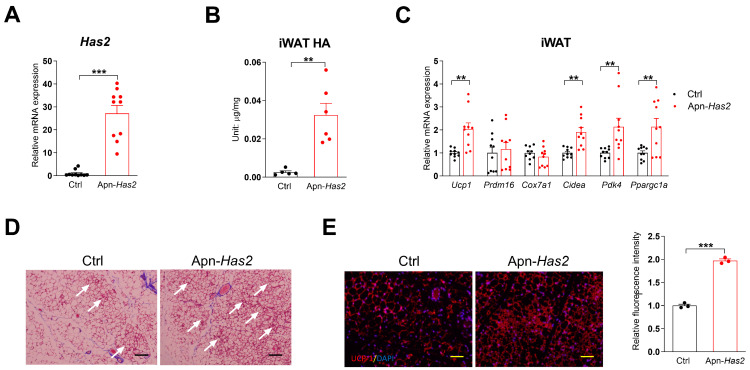
More HA enhances cold-induced iWAT beiging. (**A**) Has2 expression after 5-day Dox200 diet treatment, *n* = 10. (**B**) iWAT tissue HA levels after 5-day Dox200 diet treatment, *n* = 5–6. (**C**) Beige gene expression in iWAT harvested from mice exposed to cold and treated with Dox200 for 3 days, *n* = 10. (**D**) Representative H&E staining of iWAT from mice used in Panel (**C**), white arrows indicate the clusters of beige adipocytes. (**E**) Representative UCP-1 immunofluorescent staining of iWAT from mice used in Panel (**C**), relative fluorescence quantification of images taken from three mice is shown on the **right**. Scale bar = 100 µm. All data are mean ± SEM. ** *p* < 0.01 and *** *p* < 0.001.

Apn-rtTA/TRE-*Has2* (Apn-*Has2*) mice were generated to study how adipocyte HA production affects systemic metabolism [29]. These mice demonstrated slightly less weight gain on a high-fat diet (HFD) and significantly improved glucose tolerance [29]. Despite the lack of difference in *Ucp1* expression in BAT, Apn-*Has2* mice displayed enhanced thermogenesis when they were switched from room temperature to 6 °C in a TSE metabolic chamber [29]. After supplementing the mice with 200 mg/kg doxycycline (Dox200) in the chow diet and exposing them to cold for three days after doxycycline treatment, they displayed higher beiging gene expression (*Ucp1*, *Cidea*, *Pdk4*, and *Ppargc1a*) and morphology of more clusters of beige adipocytes (Figure 2C,D). UCP1 immunofluorescence staining also indicated higher UCP1 content in Apn-*Has2* inguinal fat pads (Figure 2E). 

### 3.3. Less HA Reduces Adipose Tissue Beiging

**Figure 3:**
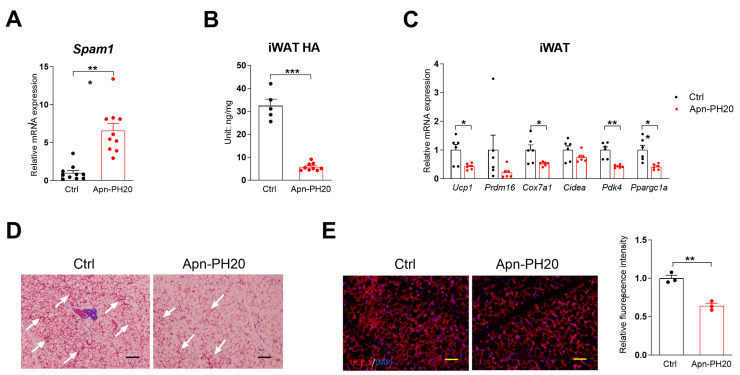
Less HA impairs cold-induced iWAT beiging. (**A**) *Spam1* expression (*n* = 10) and (**B**) iWAT tissue HA levels (*n* = 5–9) after 5-day Dox200 diet treatment. (**C**) Beige gene expression in iWAT harvested from mice exposed to cold and treated with a Dox200 diet for 3 days (*n* = 6). (**D**) Representative H&E staining of iWAT from mice used in Panel (**C**), white arrows indicate the clusters of beige adipocytes. (**E**) Representative UCP-1 immunofluorescent image of iWAT from mice used in Panel (**C**), relative fluorescence quantification of images taken from three mice is shown on the **right**. Scale bar = 100 µm. All data are mean ± SEM. * *p* < 0.05, ** *p* < 0.01, and *** *p* < 0.001.

Whether HA production itself stimulates beiging or whether secreted HA provides a beiging-enabling niche is unknown. To complement the *Has2* overexpression study, over-digestion of HA in white adipose tissue was achieved using hyaluronidase PH20. PH20 (encoded by the Spam1 gene) is a sperm-specific, secreted, glycosylphosphatidylinositol-anchored cell surface hyaluronidase that functions at neutral pH [34]. In the newly generated Apn-rtTA/TRE-PH20 (abbreviated as Apn-PH20) mice, the mRNA expression of the transgene Spam1 significantly increased (Figure 3A), while iWAT HA levels decreased by 82.2% (Figure 3B). In a similar 3-day transgene expression and cold exposure experiment, the expression of the beiging genes Ucp1, Cox7a1, Pdk4, and Ppargc1a was significantly reduced (Figure 3C). H&E staining showed fewer patches of beige adipocytes, and UCP1 immunofluorescence staining demonstrated a similar reduction (Figure 3D,E). These data suggest that digestion of HA from the extracellular space impairs cold-induced iWAT beiging.

### 3.4. HA Reprograms Gene Expression in iWAT

To understand how HA may mediate cold-induced adipose tissue beiging, we performed RNA sequence analysis of iWAT harvested from Apn-Has2 or Apn-PH20 mice compared to their respective littermate Apn-rtTA controls. Transgene expression was induced by supplementing 200 mg/kg doxycycline in the chow diet for five days in all mice, which resulted in no divergence in body weight or other metabolic phenotypes, allowing us to understand the primary effect of changes in HA levels on gene expression. 

**Figure 4:**
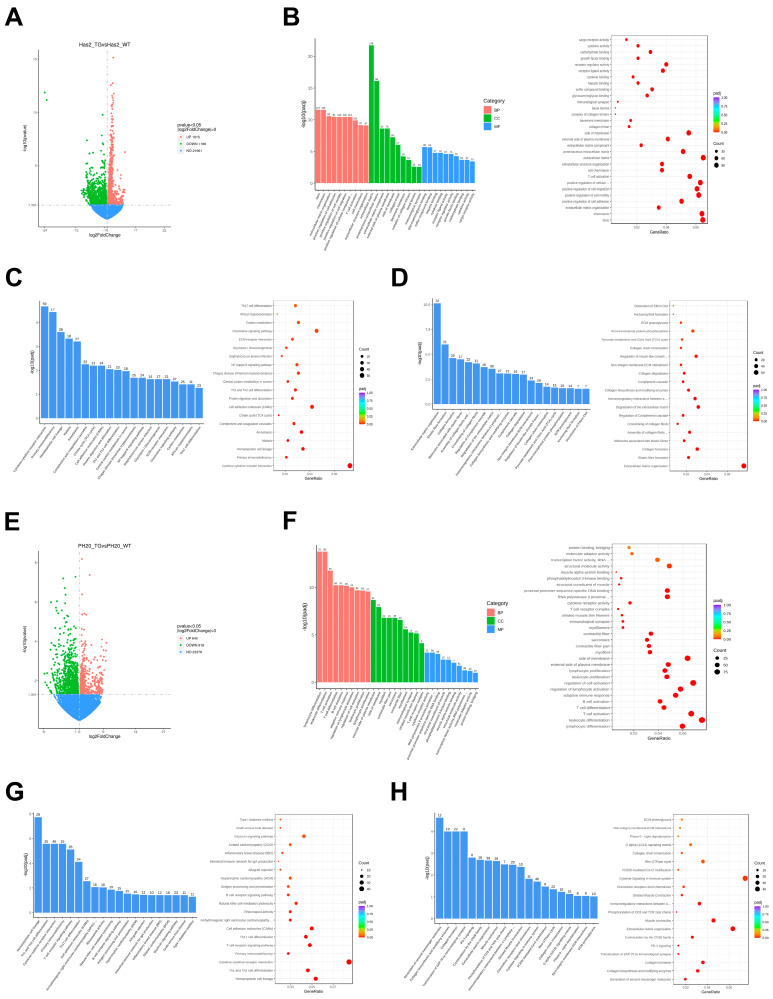

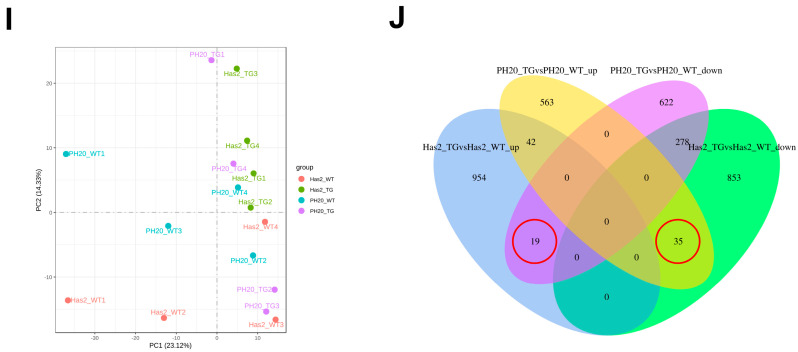
HA reprograms gene expression in iWAT. Panels (**A**–**D**): Apn-*Has2* TG mice and the control mice on a 5-day Dox 200 diet: (**A**) Differential gene volcano map. (**B**) Gene ontology (GO) enrichment analysis histogram (**left panel**) and plot of the top 30 terms (**right panel**). (**C**) KEGG enrichment results (**left panel**) and the most significant 20 KEGG pathways (**right panel**). (**D**) Reactome enrichment analysis (**left panel**) and plot of the top 20 terms (**right panel**). Panels (**E**–**H**): ApnPH20 TG mice and the control mice on a 5-day Dox 200 diet: (**E**) Differential gene volcano map. (**F**) GO enrichment analysis histogram (**left panel**) and plot of the top 30 terms (**right panel**). (**G**) KEGG enrichment results (**left panel**) and the most significant 20 KEGG pathways (**right panel**). (**H**) Reactome enrichment analysis (**left panel**) and plot of the top 20 terms (**right panel**). (**I**) Principal component analysis. (**J**) Differential expression of genes in *Has2* TG and PH20 TG animals. Changes in expression (up or down) were calculated based on the expression level of the genes comparing *Has2* overexpression (Has2 TG) or PH20 overexpression (PH20 TG) over respective AdipoqrtTA control mice.

For the Apn-Has2 study, out of 24,142 meaningful detected genes, 1015 were significantly upregulated and 1166 were significantly downregulated in the iWAT (Figure 4A). Gene ontology (GO) depicts three complementary biological concepts: biological process (BP), molecular function (MF), and cellular component (CC). For BP, taxis, chemotaxis, and extracellular matrix organization were the top three significantly changed pathways (Figure 4B), which aligns with previous studies suggesting that HA plays a role in cell migration [35]. The extracellular matrix and membrane-related pathways were the top hits in CC (Figure 4B), suggesting integrated regulation of HA levels and genes responsible for other components of the ECM [36]. Receptor-ligand activity and growth factor-binding pathways were among the top 10 MF pathways (Figure 4B), supporting the role of HA in cell proliferation [37]. KEGG enrichment pathway analysis largely supported the observation of GO analysis, highlighting cytokine–cytokine receptor interaction, chemokine signaling, and ECM–receptor interaction as the pathways with either the most significant adjusted *p*-value or highest gene ratio (Figure 4C).

Reactome enrichment showed that extracellular matrix organization was the most significantly affected pathway, while many pathways in collagen fibril trimerization, crosslinking, assembly, and degradation were among the top 20 affected pathways (Figure 4D). Notably, genes involved in collagen biosynthesis and modifying enzymes are also significantly affected by HA overproduction (Figure 4D), indicating the role of HA in ECM collagen turnover [36].

In contrast, when comparing Apn-PH20 (PH20_TG) to the ApnrtTA control (PH20_WT) iWAT, out of 24,935 meaningful detected genes, 640 genes were significantly upregulated and 919 were significantly downregulated (Figure 4E). GO pathway analysis showed that lymphocyte differentiation, leukocyte differentiation, and T cell and B cell activation were among the most significantly affected pathways (Figure 4F). The external side of the plasma membrane was the most significantly affected CM pathway, again suggesting a close relationship between HA levels and ECM components, similar to that observed in Apn-Has2 iWAT. In addition to the cytokine–receptor interaction, T cell receptor signaling pathway, hematopoietic cell lineage, and Th1 and Th2 cell differentiation pathways also play a highly significant role in KEGG pathway analysis (Figure 4G). Cytokine signaling has an important role in the immune system, and extracellular matrix organization and collagen-related pathways are again the most significant pathways in the Reactome enrichment analysis (Figure 4H). 

Principal component analysis (PCA) of the gene expression values (FPKM) of all samples showed a clear separation between Has2 WT mice and Has2 TG mice, except for Has2_WT4 (Figure 4I). However, samples in the PH20 study were dispersed and the samples within groups did not gather together (Figure 4I), suggesting a high degree of variability or heterogeneity within the group and little difference between the groups. Although several hundred to more than one thousand genes were either upregulated or downregulated by Has2 or PH20 overexpression, the Venn diagram shows that very few genes overlap and even fewer are oppositely regulated by Has2 and PH20 overexpression (Figure 4J and Table S1).

## 4. Discussion

To circumvent the low expression of β3-AR in human adipose tissue, identifying β3-AR-independent mechanisms is the key to inducing adipose tissue beiging and enhancing thermogenesis in humans. Hunting for changes in cold-induced adipose tissue beiging that are absent in CL316,243-induced adipose tissue beiging led to the discovery of the induction of HA content in iWAT only by cold exposure. Using two doxycycline-inducible mouse models with either increased or decreased iWAT HA levels, we established that HA directly promotes the white adipose tissue beiging process.

Why does cold exposure, but not CL316,243 treatment, induce HA? Cold induces sympathetic nerves in iWAT to produce and release the neurotransmitter NE, which exerts its effects by binding to and activating β-ARs located on the surface of cells to promote iWAT beiging [38]. Given that the β3-AR agonist failed to increase iWAT HA levels, β1-AR and β2-AR are suspected to mediate the cold-induced increase in iWAT HA. Previous studies have demonstrated that β1-AR and β2-AR activate adenylyl cyclase (AC) to generate the secondary messenger cyclic adenosine monophosphate (cAMP), which subsequently activates cAMP-dependent protein kinase A (PKA) and cAMP response element-binding protein (CREB) to regulate adipogenesis gene expression [39,40,41]. Bioinformatic analysis of the pCREB-binding motif indicated the possible binding of pCREB to the *Has1* and *Has2* promoters and regulatory regions, suggesting a potential regulation of HA synthesis via β1/2-ARs.

What population of cells in the adipose tissue produces HA, and how can HA promote adipose tissue beiging? Based on our unpublished data and published single-cell RNA sequencing data, we know that APCs, but not other cell types, not even adipocytes, in adipose tissue express HA synthase genes. Only APCs can produce HA in the white adipose tissue. It is well established that APCs proliferate during cold exposure, but not during CL316,243 treatment [42], and proliferating cells produce more HA [43], contributing to more HA in the iWAT of mice after cold exposure. Increased HA likely promotes APC proliferation reciprocally, given the pro-proliferative and cytoprotective properties of HA on stem cells [44,45,46]. 

The beiging process of adipose tissue can be coarsely divided into two stages: the proliferation and determination of APCs and APC differentiation into mature beige adipocytes. A larger APC pool is expected to generate more beige adipocytes when differentiation conditions permit. However, histological analysis and UCP1 staining in our animal study could not distinguish between these two stages. A lineage-tracing study coupled with the detection of proliferation using BrdU or EdU is needed to confirm this hypothesis. Notably, a recent study demonstrated that hyaluronan removal by hyaluronidase downregulates adipocyte differentiation and lipid accumulation in 3T3-L1 cells by disrupting primary cilia dynamics [47]. Given the similar differentiation process between white and beige adipocytes, it is possible that HA also promotes beige adipogenesis by enhancing the differentiation process. 

In addition to a potential link between HA levels and APC proliferation, our RNA-sequencing analysis showed a strong link between HA metabolism and immune cell development, cytokine signaling, and chemotaxis. Innate type 2 inflammation in adipose tissue promotes beiging and insulin sensitivity. Eosinophils, M2-like macrophages, and innate lymphoid type 2 cells coordinate this beiging process in adipose tissue via interleukin-33 and other interleukins [48]. HA-mediated changes in inflammation may also contribute to the control of the adipocyte beiging process.

Sex and age affect HA levels in iWAT. The female hormone estrogen is known to promote HA production in mouse skin via its receptors [49,50], and if it exerts a similar effect on APC in iWAT to promote iWAT beiging needs to be studied. On the other hand, aging is associated with a decline in the beiging potential of iWAT, and if a decrease in HA is responsible for this decline is another interesting research topic.

Finally, this study has several limitations. First, HA extraction and quantification were performed by different research personnel using different reagents over a period of several years. Owing to the difference in recovery rate, the average value of tissue HA level varied between experiments. Therefore, emphasis was placed on between-group comparisons within an experiment instead of comparing the values across different experiments. Second, there was poor overlap of differentially expressed genes in the Apn-*Has2* and Apn-PH20 RNA-seq studies. Possible explanations include the following: (1). *Has2* and PH20 overexpression may have a similar effect in depleting intracellular metabolites for HA synthesis. (2). Only genes that fall in the middle of the expression spectrum can be increased and suppressed by this type of analysis. (3). Thresholding in RNA-seq data analysis and intragroup variation also affect the statistical power and, thus, the list of overlapping differentially expressed genes. Last, a strategy of “reduction” in animal use [51] led to small sample sizes for a few experiments, potentially affecting our ability to discern statistically significant differences for certain parameters. Nevertheless, these samples provided adequate information to address the research questions.

In summary, hyaluronan levels in adipose tissue were induced by cold exposure. Elevated hyaluronan levels in adipose tissue stimulate beiging, whereas decreased hyaluronan levels suppress it. However, the exact mechanism by which HA promotes adipose tissue beiging needs further investigation.

## Figures and Tables

**Table 1:** List of primers used in the paper.

Name of the Gene	Name of the Primer	Sequence (5′ to 3′)
*H* *as2*	forward	TGTGAGAGGTTTCTATGTGTCCT
	reverse	ACCGTACAGTCCAAATGAGAAGT
*Ucp1*	forward	ACTGCCACACCTCCAGTCATT
	reverse	CTTTGCCTCACTCAGGATTGG
*Prdm16*	forward	AAGATGGAAATCGGGGAGAG
	reverse	TCTGCTTTTTGATGCAGCTC
*Cox7a1*	forward	CAGCGTCATGGTCAGTCTGT
	reverse	AGAAAACCGTGTGGCAGAGA
*Cidea*	forward	TGCTCTTCTGTATCGCCCAGT
	reverse	GCCGTGTTAAGGAATCTGCTG
*Pdk4*	forward	GCCGTGTTAAGGAATCTGCTG
	reverse	TCTACAAACTCTGACAGGGCTTT
*Ppargc1a*	forward	AGCCGTGACCACTGACAACGAG
	reverse	GCTGCATGGTTCTGAGTGCTAAG
*Spam1*	forward	CCAGACGACAAATTGGGCTTA
	reverse	TCCTGGATTAGTTGATTGGACCA
*Rps16*	forward	GATTTGCTGGTGTGGATATT
	reverse	TCTTTGATCTCCTTCTTGGA

## Data Availability

The gene expression data generated from RNA-seq analysis were deposited in the Gene Expression Omnibus (GEO) database under accession number GSE268781. All other data supporting the findings of this study are available upon reasonable request. The mouse models are available from the corresponding author upon request.

## Supplementary Materials

The following supporting information can be downloaded at: https://www.mdpi.com/article/10.3390/cells13151233/s1, Figure S1: Serum and iWAT HA levels in male vs. female and young vs. old mice.

## References

[1] Wang Q.A., Tao C., Gupta R.K., Scherer P.E. (2013). Tracking adipogenesis during white adipose tissue development, expansion and regeneration. Nat. Med..

[2] Petrovic N., Walden T.B., Shabalina I.G., Timmons J.A., Cannon B., Nedergaard J. (2010). Chronic peroxisome proliferator-activated receptor gamma (PPARgamma) activation of epididymally derived white adipocyte cultures reveals a population of thermogenically competent, UCP1-containing adipocytes molecularly distinct from classic brown adipocytes. J. Biol. Chem..

[3] Wu J., Bostrom P., Sparks L.M., Ye L., Choi J.H., Giang A.H., Khandekar M., Virtanen K.A., Nuutila P., Schaart G. (2012). Beige adipocytes are a distinct type of thermogenic fat cell in mouse and human. Cell.

[4] Pollard A.E., Carling D. (2020). Thermogenic adipocytes: Lineage, function and therapeutic potential. Biochem. J..

[5] Wang C.H., Wei Y.H. (2021). Therapeutic Perspectives of Thermogenic Adipocytes in Obesity and Related Complications. Int. J. Mol. Sci..

[6] Lizcano F. (2019). The Beige Adipocyte as a Therapy for Metabolic Diseases. Int. J. Mol. Sci..

[7] Cypess A.M., Lehman S., Williams G., Tal I., Rodman D., Goldfine A.B., Kuo F.C., Palmer E.L., Tseng Y.H., Doria A. (2009). Identification and importance of brown adipose tissue in adult humans. N. Engl. J. Med..

[8] van Marken Lichtenbelt W.D., Vanhommerig J.W., Smulders N.M., Drossaerts J.M., Kemerink G.J., Bouvy N.D., Schrauwen P., Teule G.J. (2009). Cold-activated brown adipose tissue in healthy men. N. Engl. J. Med..

[9] Virtanen K.A., Lidell M.E., Orava J., Heglind M., Westergren R., Niemi T., Taittonen M., Laine J., Savisto N.J., Enerback S. (2009). Functional brown adipose tissue in healthy adults. N. Engl. J. Med..

[10] Sidossis L.S., Porter C., Saraf M.K., Borsheim E., Radhakrishnan R.S., Chao T., Ali A., Chondronikola M., Mlcak R., Finnerty C.C. (2015). Browning of Subcutaneous White Adipose Tissue in Humans after Severe Adrenergic Stress. Cell Metab..

[11] Baskin A.S., Linderman J.D., Brychta R.J., McGehee S., Anflick-Chames E., Cero C., Johnson J.W., O’Mara A.E., Fletcher L.A., Leitner B.P. (2018). Regulation of Human Adipose Tissue Activation, Gallbladder Size, and Bile Acid Metabolism by a beta3-Adrenergic Receptor Agonist. Diabetes.

[12] Cypess A.M., Weiner L.S., Roberts-Toler C., Franquet Elia E., Kessler S.H., Kahn P.A., English J., Chatman K., Trauger S.A., Doria A. (2015). Activation of human brown adipose tissue by a beta3-adrenergic receptor agonist. Cell Metab..

[13] O’Mara A.E., Johnson J.W., Linderman J.D., Brychta R.J., McGehee S., Fletcher L.A., Fink Y.A., Kapuria D., Cassimatis T.M., Kelsey N. (2020). Chronic mirabegron treatment increases human brown fat, HDL cholesterol, and insulin sensitivity. J. Clin. Investig..

[14] Sharp L.Z., Shinoda K., Ohno H., Scheel D.W., Tomoda E., Ruiz L., Hu H., Wang L., Pavlova Z., Gilsanz V. (2012). Human BAT possesses molecular signatures that resemble beige/brite cells. PLoS ONE.

[15] Jiang Y., Berry D.C., Graff J.M. (2017). Distinct cellular and molecular mechanisms for beta3 adrenergic receptor-induced beige adipocyte formation. Elife.

[16] Shao M., Wang Q.A., Song A., Vishvanath L., Busbuso N.C., Scherer P.E., Gupta R.K. (2019). Cellular Origins of Beige Fat Cells Revisited. Diabetes.

[17] Arch J.R. (2008). The discovery of drugs for obesity, the metabolic effects of leptin and variable receptor pharmacology: Perspectives from beta3-adrenoceptor agonists. Naunyn Schmiedebergs Arch. Pharmacol..

[18] Michel M.C., Ochodnicky P., Summers R.J. (2010). Tissue functions mediated by beta(3)-adrenoceptors-findings and challenges. Naunyn Schmiedebergs Arch. Pharmacol..

[19] Schena G., Caplan M.J. (2019). Everything You Always Wanted to Know about beta3-AR * (* But Were Afraid to Ask). Cells.

[20] Riis-Vestergaard M.J., Richelsen B., Bruun J.M., Li W., Hansen J.B., Pedersen S.B. (2020). Beta-1 and Not Beta-3 Adrenergic Receptors May Be the Primary Regulator of Human Brown Adipocyte Metabolism. J. Clin. Endocrinol. Metab..

[21] Evans B.A., Merlin J., Bengtsson T., Hutchinson D.S. (2019). Adrenoceptors in white, brown, and brite adipocytes. Br. J. Pharmacol..

[22] Li Y., Ping X., Zhang Y., Li G., Zhang T., Chen G., Ma X., Wang D., Xu L. (2021). Comparative Transcriptome Profiling of Cold Exposure and beta3-AR Agonist CL316,243-Induced Browning of White Fat. Front. Physiol..

[23] Mo X., Liu E., Huang Y. (2019). The intra-brain distribution of brain targeting delivery systems. Brain Targeted Drug Delivery System.

[24] Frantz C., Stewart K.M., Weaver V.M. (2010). The extracellular matrix at a glance. J. Cell Sci..

[25] Zhu Y., Crewe C., Scherer P.E. (2016). Hyaluronan in adipose tissue: Beyond dermal filler and therapeutic carrier. Sci. Transl. Med..

[26] Zhu Y., Kruglikov I.L., Akgul Y., Scherer P.E. (2018). Hyaluronan in adipogenesis, adipose tissue physiology and systemic metabolism. Matrix Biol..

[27] Laurent T.C., Laurent U.B., Fraser J.R. (1996). The structure and function of hyaluronan: An overview. Immunol. Cell Biol..

[28] Fraser J.R., Laurent T.C., Laurent U.B. (1997). Hyaluronan: Its nature, distribution, functions and turnover. J. Intern. Med..

[29] Zhu Y., Li N., Huang M., Bartels M., Dogne S., Zhao S., Chen X., Crewe C., Straub L., Vishvanath L. (2021). Adipose tissue hyaluronan production improves systemic glucose homeostasis and primes adipocytes for CL 316,243-stimulated lipolysis. Nat. Commun..

[30] Zhu Y., Zhao S., Deng Y., Gordillo R., Ghaben A.L., Shao M., Zhang F., Xu P., Li Y., Cao H. (2017). Hepatic GALE Regulates Whole-Body Glucose Homeostasis by Modulating Tff3 Expression. Diabetes.

[31] Wang X., Spandidos A., Wang H., Seed B. (2012). PrimerBank: A PCR primer database for quantitative gene expression analysis, 2012 update. Nucleic Acids Res.

[32] Tian X., Azpurua J., Hine C., Vaidya A., Myakishev-Rempel M., Ablaeva J., Mao Z., Nevo E., Gorbunova V., Seluanov A. (2013). High-molecular-mass hyaluronan mediates the cancer resistance of the naked mole rat. Nature.

[33] Danysz W., Jinlai K., Li F. (2019). Duration of a “Brown-Like” Phenotype of White Adipose Tissue Induced by the beta3 Agonist CL-316,243. Drug Res..

[34] Reitinger S., Laschober G.T., Fehrer C., Greiderer B., Lepperdinger G. (2007). Mouse testicular hyaluronidase-like proteins SPAM1 and HYAL5 but not HYALP1 degrade hyaluronan. Biochem. J..

[35] Solis M.A., Chen Y.H., Wong T.Y., Bittencourt V.Z., Lin Y.C., Huang L.L. (2012). Hyaluronan regulates cell behavior: A potential niche matrix for stem cells. Biochem. Res. Int..

[36] Evanko S.P., Potter-Perigo S., Petty L.J., Workman G.A., Wight T.N. (2015). Hyaluronan Controls the Deposition of Fibronectin and Collagen and Modulates TGF-beta1 Induction of Lung Myofibroblasts. Matrix Biol..

[37] Dovedytis M., Liu Z.J., Bartlett S. (2020). Hyaluronic acid and its biomedical applications: A review. Eng. Regen..

[38] Blaszkiewicz M., Willows J.W., Johnson C.P., Townsend K.L. (2019). The Importance of Peripheral Nerves in Adipose Tissue for the Regulation of Energy Balance. Biology.

[39] Delghandi M.P., Johannessen M., Moens U. (2005). The cAMP signalling pathway activates CREB through PKA, p38 and MSK1 in NIH 3T3 cells. Cell. Signal..

[40] Carlezon W.A., Duman R.S., Nestler E.J. (2005). The many faces of CREB. Trends Neurosci..

[41] Herzig S., Long F., Jhala U.S., Hedrick S., Quinn R., Bauer A., Rudolph D., Schutz G., Yoon C., Puigserver P. (2001). CREB regulates hepatic gluconeogenesis through the coactivator PGC-1. Nature.

[42] Oguri Y., Shinoda K., Kim H., Alba D.L., Bolus W.R., Wang Q., Brown Z., Pradhan R.N., Tajima K., Yoneshiro T. (2020). CD81 Controls Beige Fat Progenitor Cell Growth and Energy Balance via FAK Signaling. Cell.

[43] Asselman M., Verhulst A., Van Ballegooijen E.S., Bangma C.H., Verkoelen C.F., De Broe M.E. (2005). Hyaluronan is apically secreted and expressed by proliferating or regenerating renal tubular cells. Kidney Int..

[44] Chen P.Y., Huang L.L., Hsieh H.J. (2007). Hyaluronan preserves the proliferation and differentiation potentials of long-term cultured murine adipose-derived stromal cells. Biochem. Biophys. Res. Commun..

[45] Zhao N., Wang X., Qin L., Guo Z., Li D. (2015). Effect of molecular weight and concentration of hyaluronan on cell proliferation and osteogenic differentiation in vitro. Biochem. Biophys. Res. Commun..

[46] Luo Y., Liang F., Wan X., Liu S., Fu L., Mo J., Meng X., Mo Z. (2022). Hyaluronic Acid Facilitates Angiogenesis of Endothelial Colony Forming Cell Combining with Mesenchymal Stem Cell via CD44/MicroRNA-139-5p Pathway. Front. Bioeng. Biotechnol..

[47] Drygalski K., Higos R., Merabtene F., Mojsak P., Grubczak K., Ciborowski M., Razak H., Clement K., Dugail I. (2024). Extracellular matrix hyaluronan modulates fat cell differentiation and primary cilia dynamics. Biochim. Biophys. Acta Mol. Cell Biol. Lipids.

[48] Bolus W.R., Hasty A.H. (2019). Contributions of innate type 2 inflammation to adipose function. J. Lipid Res..

[49] Uzuka M., Nakajima K., Ohta S., Mori Y. (1981). Induction of hyaluronic acid synthetase by estrogen in the mouse skin. Biochim. Biophys. Acta.

[50] Uzuka M., Nakajima K., Ohta S., Mori Y. (1980). The mechanism of estrogen-induced increase in hyaluronic acid biosynthesis, with special reference to estrogen receptor in the mouse skin. Biochim. Biophys. Acta.

[51] Institute of Laboratory Animal Resources (US) (2011). Guide for the Care and Use of Laboratory Animals.

