# Supplementary material for: Hyaluronan Mediates Cold-Induced Adipose Tissue Beiging
Source: Cells. 2024 Jul 23;13(15):1233. doi: 10.3390/cells13151233 (PMC11311271; doi:10.3390/cells13151233)
Supplement: Supplementary file 1 [file cells-13-01233-s001.zip › Supplementary Figures.pdf]

Supplementary figures

S1

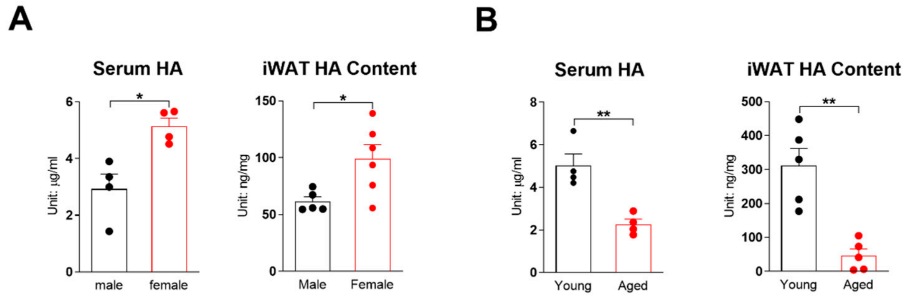

Supplementary Figure S1. Serum and iWAT HA levels in male vs female and young vs old mice. Serum HA concentration and iWAT HA content in **(A)** male or female mice at the age of 20 weeks, and **(B)** in young (10 weeks) or old (75 weeks) male mice; n = 4 - 6. All data are mean ± SEM. *$p < 0.05$, **$p < 0.01$.
